# Supplementary material for: Dataset of single nucleotide polymorphisms of immune-associated genes in patients with SARS-CoV-2 infection
Source: PLoS One. 2023 Nov 16;18(11):e0287725. doi: 10.1371/journal.pone.0287725 (PMC10653545; doi:10.1371/journal.pone.0287725)
Supplement: S1 Fig — The prediction of SNP rs993496436 is benign. The SNP causes a residue change from serine to cysteine at position 190 in the amino acid chain. (PDF) [file pone.0287725.s001.pdf]

PolyPhen-2 report for Q9Y286 S190C

Query

Protein Acc	Position	AA_1	AA_2	Description
Q9Y286	190	S	C	Canonical; RecName: Full=Sialic acid-binding Ig-like lectin 7; Short=Siglec-7; AltName: Full=Adhesion inhibitory receptor molecule 1; Short=AIRM-1; AltName: Full=CDw328; AltName: Full=D-siglec; AltName: Full=QA79 membrane protein; AltName: Full=p75; AltName: CD_antigen=CD328; Flags: Precursor; Length: 467

Results

Prediction/Confidence *PolyPhen-2 v2.2.2r406*

HumDiv

This mutation is predicted to be **BENIGN** with a score of **0.447** (sensitivity: **0.89**; specificity: **0.90**)

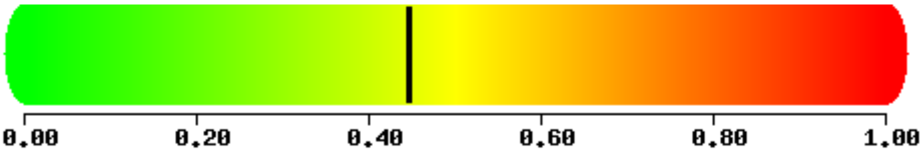

HumVar

This mutation is predicted to be **BENIGN** with a score of **0.309** (sensitivity: **0.86**; specificity: **0.77**)

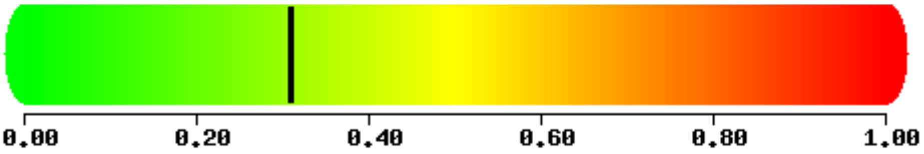

Details

Multiple sequence alignment *UniProtKB/UniRef100 Release 2011_12 (14-Dec-2011)*

3D Visualization *PDB/DSSP Snapshot 25-May-2021 (178229 Structures)*
