# Supplementary material for: Dataset of single nucleotide polymorphisms of immune-associated genes in patients with SARS-CoV-2 infection
Source: PLoS One. 2023 Nov 16;18(11):e0287725. doi: 10.1371/journal.pone.0287725 (PMC10653545; doi:10.1371/journal.pone.0287725)
Supplement: Attachment — Submitted filename: Covid_Manuscript_Final-v2.docx [file pone.0287725.s003.docx]

# Dataset of single nucleotide polymorphisms of immune-associated genes in patients with SARS-CoV2 infection

Nikoletta Katsaouni^1,2,3,#^, Pablo Llavona^4#^, Yascha Khodamoradi^5#^, Ann-Kathrin Otto^6^, Stephanie Körber^7^, Christof Geisen^7^, Christian Seidl^7^, Maria J. G. T. Vehreschild^5^, Sandra Ciesek^8,9,10^, Jörg Ackermann^6^, Ina Koch^6^, Marcel H. Schulz^1,2,3,§*^, Daniela S. Krause^4,7,11,12,13§*^

^1^Computational Epigenomics & Systems Cardiology, Institute of Cardiovascular Regeneration, Goethe University and University Clinic, Frankfurt am Main, Germany.

^2^German Center for Cardiovascular Research (DZHK), Partner site RheinMain, Frankfurt am Main, Germany

^3^Cardio-Pulmonary Institute, Goethe University Hospital, Frankfurt am Main, Germany

^4^Georg-Speyer-Haus, Institute for Tumor Biology and Experimental Therapy, Frankfurt am Main, Germany.

^5^Department of Internal Medicine, Infectious Diseases, University Hospital Frankfurt, Goethe University Frankfurt, Germany

^6^Molecular Bioinformatics, Institute of Computer Science, Goethe University Frankfurt am Main, Germany

^7^German Red Cross Blood Donor Service Baden-Württemberg Hessen, Frankfurt am Main, Germany.

^8^Institute for Medical Virology, University Hospital, Goethe University

Frankfurt

^9^German Centre for Infection Research, External Partner Site, Frankfurt, Germany.

^10^Fraunhofer Institute for Molecular Biology and Applied Ecology (IME), Branch Translational Medicine and Pharmacology, Frankfurt, Germany.

^11^Faculty for Biochemistry II and Institute of General Pharmacology and Toxicology, Goethe-University, Frankfurt am Main, Germany.

^12^German Cancer Consortium (DKTK), Germany.

^13^Frankfurt Cancer Institute, Frankfurt am Main, Germany.

^#^These authors contributed equally.

^§^These authors contributed equally.

Corresponding authors:

*Prof. Dr. Daniela S. Krause

Goethe-University Frankfurt

c/o Georg-Speyer-Haus

Paul-Ehrlich-Str. 42-44

60596 Frankfurt am Main

Phone: +49 69 63395 500

Fax: +49 69 63395 519

Email: [Krause@gsh.uni-frankfurt.de](mailto:Krause@gsh.uni-frankfurt.de)

*Prof. Dr. Marcel H. Schulz

Goethe-University Frankfurt

Institute for Cardiovascular Regeneration

Theodor-Stern-Kai 7

60590 Frankfurt am Main

Phone: +49 69 6301 86203

Email: [marcel.schulz@em.uni-frankfurt.de](mailto:marcel.schulz@em.uni-frankfurt.de)

### Abstract

The SARS-CoV2 pandemic has affected nations globally leading to illness, death and economic downturn. Why disease severity, ranging from no symptoms to the requirement for extracorporeal membrane oxygenation, varies between patients is still incompletely understood. Consequently, we aimed at understanding the impact of genetic factors to disease severity in infection with SARS-CoV2. Here, we provide data on demographics, ABO blood group, human leukocyte antigen (HLA) type, as well as next-generation sequencing data of genes in the natural killer cell receptor family, the renin-angiotensin-aldosterone and kallikrein-kinin systems and others in 159 patients with SARS-CoV2 infection, stratified into seven categories of disease severity. We provide single-nucleotide polymorphism (SNP) data on the patients and a protein structural analysis as a case study on a SNP in the *SIGLEC7* gene, which was significantly associated with clinical score. Our data represent a resource for correlation analyses involving genetic factors and disease severity and may help predict outcome in infections with future SARS-CoV2 variants and aid vaccine adaptation.

### Background and Summary

The pandemic due to severe acute respiratory syndrome coronavirus 2 (SARS-CoV-2) has devastatingly affected the entire globe causing disease and death to millions, as well as unemployment, social hardship and a shrinkage of global growth, which represents the deepest recession since World War II [1, 2]. While vaccines have become available in industrialized nations and have favorably impacted infection rates, continuously evolving mutations in the viral genome are leading to new waves of infection and causing vaccines to become less efficient [3].

Symptoms of infection with SARS-CoV-2 vary and range from no or mild symptoms, such as headaches, nasal congestion, fever, muscle pain, sore throat and a loss of taste and smell, to severe illness. In severe cases dyspnea, hypoxia and lung involvement, which may lead to respiratory failure, shock and multi-organ dysfunction, requiring ventilation or extracorporeal membrane oxygenation have been observed [1].

Approximately 80% of patients with SARS-CoV-2 infection recover from the disease without the need for treatment, while approximately 5% are admitted to intensive care, 2.3% require mechanical ventilation and 1.4% of patients die [4]. Advanced age and comorbidities such as diseases of the cardiovascular system, chronic pulmonary diseases, diabetes, cancer and immunosuppression have been considered risk factors for a severe clinical course or fatal outcome [5]. It is, however, unknown why even certain younger, otherwise healthy individuals or members of certain ethnic groups, may experience severe or even fatal disease or a condition characterized by fatigue and other symptoms termed long COVID.

Since the outbreak of the pandemic, various publications have described polymorphisms in certain genes or variations in expression levels of proteins to influence disease severity and outcome in SARS-CoV-2 infection. Prominent examples are the solute carrier family 6 member 20 (SLC6A20) [1], sialic acid-binding Ig-like lectin 7 (SIGLEC7; CD328) [6], and transmembrane serine protease 2 (TMPRSS2) [7]. SLC6A20 is a member of the family of Na^+^ and Cl^-^-coupled transporters expressed by proximal tubule kidney cells (and enterocytes), where they function as transporters of proline [8, 9]. SLC6A20 has been identified as part of a 3p21.31 gene cluster representing a genetic susceptibility locus in SARS-CoV-2 patients with respiratory failure [1]. By hetero-dimerization with angiotensin converting enzyme (ACE)2, SLC6A20 is associated with the renin-angiotensin system. In the renin-angiotensin system, ACE2 plays a key role in the conversion of the vasoconstrictor angiotensin-II (Ang-II) into the vasodilator Ang1-7 and, thereby, a lowering of the blood pressure [10]. TMPRSS2 promotes the uptake of SARS-CoV-2 via proteolytic cleavage of the ACE2 receptor. Single nucleotide polymorphisms in TMPRSS2 have been shown to vary amongst global populations [7] and to affect the susceptibility of a patient to infection with SARS-CoV-2 via modulation of splicing, miRNA expression etc. [11]. SIGLEC7 facilitates the inhibition of the cytotoxicity of natural killer cells [12]. Expression of SIGLEC7 has been shown to correlate with SARS-CoV-2 levels in nasopharyngeal swabs or autopsies [6].

Based on this previous findings, we collected demographics, ABO blood group, human leukocyte antigen (HLA) type, data on hereditary prothrombotic factors, as well as next-generation sequencing data of genes in the natural killer cell receptor family, the renin-angiotensin-aldosterone and kallikrein-kinin systems in patients. Patients had been stratified into seven categories of disease severity according to the World Health Organization (WHO) Ordinal Scale for Clinical Improvement.

Our data may aid predictions on the susceptibility of individuals to novel virus variants, their anticipated disease severity, as well as on the efficiency of current and future vaccines to viral mutants. It is hoped that, our data will support the rational design, adaptation and optimization of vaccines to new viral strains.

### Methods

#### Patient collection

10 ml of peripheral blood were collected in EDTA from 159 patients, who had been or were currently infected with SARS-CoV2 and who received medical care from the university hospital of the Goethe-University in Frankfurt am Main, Germany. All patient samples were collected between April 2020 and November 2021, a period when the virus strains B.1.1.7 and B.1.617 or B.1.617.2 were most prevalent. All included patients were tested positive by polymerase chain reaction (PCR) at different institutions. All human patients gave informed consent to their participation in the study according to the Declaration of Helsinki. The ethics committee of the Goethe University Frankfurt had previously approved the study. DNA for HLA typing was available for 156 of the patients. 67 patients were female (42 %), 90 patients were male (56 %), and two patients were not reported. SNP sequencing was conducted for all patients. The patients were between 18-92 years of age. The patients were stratified according to the World Health Organization Ordinal Scale for Clinical Improvement [13], with a

- score 1 signifying very mild symptoms without compromise of activities,
- score 2 signifying mild symptoms with compromise of activities,
- score 3 signifying moderate disease requiring admission to hospital (but no oxygen),
- score 4 signifying hospital care with oxygen treatment,
- score 5 signifying severe disease with high oxygen requirements,
- score 6 signifying intubation and mechanical ventilation and
- score 7 signifying ventilation plus support of organs by pressors or extracorporeal membrane oxygenation (ECMO).

The scoring system for the patients was compared to an updated scoring system [14]. For further data and future statistical analysis, patients were categorized as mild (≤2, n=94) or severe disease (≥3, n=65), with the class ‘severe disease’ defining patients who were hospitalized. Respiratory hospitalization was defined as hospitalization due to dyspnea requiring oxygen treatment, mechanical ventilation or ECMO (scores ≥4, n=51). The steps of the experimental workflow can be found in Figure 1.

Table 1 (Excel version online available at <https://doi.org/10.6084/m9.figshare.21803928.v1>) lists the gender, age, clinical score, ABO type, Rhesus type, HLA types, ethnicity, and clinical information on comorbidities for each individual patient. Information on the classification of the patients according to the WHO Clinical Improvement clinical progression scale [14] is provided and can be found in column “Clinical Score”.

Table 1: Clinical metadata and HLA types for each individual patient in the cohort of 159 patients. Note that, sample IDs 33, 45, and 79 are not included. Excel version is online available at <https://doi.org/10.6084/m9.figshare.21803928.v1>.

#### Blood typing

Blood typing for A, B, O was performed using the Bioclone Kit from Ortho-Clinical Diagnostics (Unterschleissheim, Germany) according to the manufacturer’s instructions. Rhesus factor was determined using the IgM Anti-D Mono-Type reagent from Medion Grifols Diagnostic AG (Düdingen, Switzerland).

#### DNA isolation

Red blood cells (RBCs) were lysed with ACK lysis buffer (Thermo Fisher, Waltham, MA). The remaining cells were washed and centrifuged (at 1780 x g for 15 min), before they were collected in resuspension buffer (NaCl 6M, Na_2_EDTA pH 8), to which proteinase K (10 mg/ml) and 20% w/v sodium dodecyl sulfate (SDS) solution for overnight cell lysis at 37 $^{\circ}$C had been added. Cell debris was separated from nucleic acids after incubation in 6 M NaCl at 55 $^{\circ}$C for 15 min. The DNA was purified with 96% and 70% ethanol and finally re-suspended in TE buffer.

#### HLA genotyping

Next generation sequencing for HLA-A, -B, -C, -DRB1, -DQB1 and -DPB1 was performed by a high-resolution amplicon-based approach using NGSgo-MX6- and NGSgo-LibrX kits (GENDX, Utrecht, The Netherlands) according to the manufacturer’s instructions on an Illumina Mini-Seq device (Illumina, San Diego, CA).

#### Genotyping for Factor V Leiden and Prothrombin G20210A

The Factor V Leiden and Prothrombin (20210G>A)-genotypes, in the HGVS nomenclature version 20.05 NM000130.4: c.1601G>A, p.(Arg534Gln) and NM_000506.4: c.*97G>A, respectively, were determined by sequence-specific PCR (Factor V Leiden Quicktype and Factor II 20210 G>A Quicktype, Attomol Molekulare Diagnostika GmbH, Bronkow, Germany) according to the manufacturer’s instructions. The samples were analyzed in a 3% agarose gel in comparison to a blank, a negative (wildtype DNA) and a heterozygous control.

#### Design of custom-made next-generation sequencing (NGS) panel

A customized NGS panel was designed to cover exonic and partially intronic regions within genes in the natural killer cell receptor family [15], the renin-angiotensin-aldosterone system, the kallikrein-kinin system and other genes previously found to be relevant in SARS-CoV2-infection [1]. In total, several genomic loci, spanning 144,830 bp, were targeted by sequencing. Overall, the custom-made NGS panel targeted the coding, untranslated and splicing-regulatory intronic regions of 90 genes. For a list of the genes, we refer to Figure 2. Primer sets for the targeted regions were designed *de novo* by Illumina DesignStudio.

#### NGS library preparation and sequencing (non-HLA sequencing)

The DNA library preparation was performed following the Illumina DNA Prep with Enrichment protocol (Illumina, San Diego, USA) according to the manufacturer’s instructions. 600 ng of genomic input DNA were used for the generation of the libraries. The libraries were purified with AMPure XP beads (Beckman Coulter, High Wycombe, UK). Additionally, libraries and sample pools were quantified using Qubit dsDNA HS kit and Qubit 4 (Thermo Fisher Scientific, Waltham, USA), whereas the library size was analyzed with High Sensitivity D1000 assay and 4200 Tapestation (Agilent Technologies, Palo Alto, USA). Samples were pooled reaching a final 1.4 pM library solution, which, subsequently, was loaded on the Illumina MiniSeq platform (Illumina, San Diego, USA) for (150 bp x 2) paired-end sequencing.

#### Read analysis and variant calling

Reads generated from the targeted regions, were aligned to the reference genome hg19/GRCh37 using the Burrows Wheeler Aligner (0.7.7-isis-1.0.2) [16]. Generation of indexed Sequence Alignment/Map (SAM) and its Binary Alignment/Map (BAM) file versions were computed using SAMtools (0.1.19-isis-1.0.3). Variants were called and annotated using the GATK package (v1.6-23-gf0210b3) [17]. Sequence data were processed using the GensearchNGS software suit (1.7.058, Phenosystems, Braine le Chateau, Belgium). Only samples with a mean coverage depth higher than 90 on-target passing-filter reads were considered for subsequent analysis. Data were filtered based on a variant coverage of at least ten reads and a minimum variant allele frequency (VAF) of 20%. The detected variants were assessed with the dbSNP database [18] (The Genome Aggregation Database, NHLBI Exome Sequencing Project, NCBI dbSNP, Human Gene Mutation Database). Further, variant *in silico* prediction was carried out with PolyPhen-2 [19].

#### Association testing

The focus of our analyses were the genes *SIGLEC7*, *ACE*, *SELL*, *TMPRSS2,* and *SLC6A20*, since they were found to be associated with COVID-19 severity in recent studies [1][6, 7]. To explore SNPs, which lead to increased severity and to find novel SNPs, we conducted an association analysis.

SNPs that occurred in less than four patients were filtered out and excluded by the analysis. Testing significance, the p-values for the SNPs were calculated using logistic regression and adjusted for covariates gender and age. The open-source whole-genome association analysis toolset PLINK (version 1.9) [19] was used to build the regression model. To adjust for multiple testing, the p-values were corrected using false discovery rate approach by Benjamini-Hochberg [20].

To consider associations between all the variants within each gene, gene-based tests were applied. Accounting for multiple independent functional variants may increase the power to identify disease-associated genes. By aggregating the information on all the variants, we obtained a single p-value which corresponds to the significance of association of the gene. We applied the versatile gene-based test for genome-wide association studies (VEGAS) [21] and the gene-based association test with extended Simes procedure (GATES) [22]. Concerning the role of HLA genotypes, the dataset limits power for the calculation of significant association, similar to the statistical analysis by Schetelig *et al*. [23].

#### Analysis of mutation impact on protein structure

To find the SNP-containing structures in the Protein Data Bank (PDB) [24], we used UniProt [25]. We applied the SWISS-MODEL repository and the related analysis and visualization tool [26] to check the structural coverage. For unknown structures and regions without structural coverage, we analyzed predicted structures based on the AlphaFold database [27, 28]. For each predicted structure, AlphaFold provides a color-coded confidence score per residue to evaluate the prediction quality. To estimate the impact of a SNP on protein structure and function we ran the PolyPhen-2 software [19].

PolyPhen-2 annotates coding, nonsynonymous SNPs and predicts damaging missense mutations, using sequence-based and structure-based predictive features. To train and test PolyPhen-2, two pairs of datasets were generated. The first pair, HumDiv, is based on all 3,155 damaging alleles annotated in UniProt as causing human Mendelian diseases and affecting protein stability or function, as well as on 6,321 differences between human proteins and their closely related mammalian homologs, which have been assumed to be non-damaging. The second pair, HumVar3, consists of all the 13,032 human disease-causing mutations from UniProt and 8,946 human nonsynonymous single-nucleotide polymorphisms (nsSNPs) without annotated involvement in disease, which have been treated as non-damaging. PolyPhen-2 qualitatively classifies a mutation as benign, possibly damaging, or probably damaging, scoring with a value between 0 and 1, with 1 for damaging impact and 0 for non-damaging impact of the SNP on the protein structure.

#### **Integration of other studies**

32 SNPs of five genes of interest, ACE, SIGLEC7, SELL, TMPRSS2, and SLC6A20, were rare variants and not reported in other datasets such as gnomAD (version 2.1.1) [29] or dbSNP. For a list of the 32 rare variants, we refer to Figure 2.

### Results

#### **Patient cohort**

We gathered a dataset of patients with SARS-CoV2 infections, for an outline of the experimental workflow and sequencing, we refer to Figure 1. Figure 2 lists 32 SNPs of the five genes *ACE*, *SIGLEC7*, *SELL*, *TMPRSS2,* and *SLC6A20* that are rare variants and not reported preciously in other datasets such as gnomAD. Figure 3 depicts distributions of age, clinical score, ABO blood type, and HLA type for the patient cohort. We found no statistical significance for clinical scores of patients of any subgroup of ABO blood type or HLA type, i.e., Mann-Whitney U tests gave significances values larger than 5%.

Figure 4 illustrates the role of age and gender for the clinical score. In Jitter plot Figure 4A the red and blue dots represent data points for female and male patients, respectively. The grey curve, unisex, shows the increase of the mean clinical score for all patients. The mean clinical score increases with age. High clinical score is associated with high age (Kendall tau 0.41, p= 1.2 10^-12^, Stuart-Kendall Tau-c test). The unisex curve has its maximal slope of 0.1874 +/- 0.0495 [clinical score / year] at age 60.394 +/- 3.466 years. The blue curve, male, shows the increase of the mean clinical score for male patients. The male curve has its maximal slope of 0.245 +/- 0.124 [clinical score / year] at age 55.9 +/- 2.8 years. High clinical score of male patients is associated with high age (Kendall tau 0.41, p= 1.2 10^-7^, Stuart-Kendall Tau-c test). The red curve, female, shows the increase of mean clinical score for female patients. The female curve is steeper than the male curve and has its maximal slope of 0.942 +/- 0.552 [clinical score / year] at age 66.1 +/- 1.0 years. High clinical score of female patients is associated with high age (Kendall tau 0.34, p= 1.0 10^-4^, Stuart-Kendall Tau-c test). For young (age < 40) and old (age > 70), the clinical scores of female and male patients were not significantly different (Mann–Whitney U test). For age > 50 years, the mean clinical score increases monotonically with age, see Figure 4B, and the clinical scores differ significantly from younger patients (age < 50, Mann–Whitney U test). The clinical scores of females are significantly lower than for males (Mann-Whitney U test, p-value 1.02E-4), see Figure 4C. Figure 4D shows the receiver operating characteristics (ROC) of a random forest prediction of hospitalization, i.e., clinical score 4-7, based on age and gender. The blue line is the mean ROC curve of a Monte Carlo cross-validation with 100 random splits into 70% training and 30% test sets. The shaded area, light gray, indicates the standard deviation. The mean area under the curve (AUC) value of 0.72± 0.08 demonstrates the power of age and gender in predicting hospitalization of a patient. For a discussion of the role of gender and age on Covid-19 severity, we refer to [31].

#### **Panel sequencing quality**

On average more than 1.2 M of passing-filter (PF) reads were generated per sample with 89.1% of them in Q3. Similarly, more than 1.2 M reads aligned to the reference genome representing 98.7 % of all PF reads, generating a mean coverage of 332.6x per sample with a coverage uniformity of 98.7%.

Sample sequence-containing and base quality score files (FastQ) were aligned to the reference genome and transformed into SAM files and its binary counterpart, BAM files. The latter were subjected to variant calling and annotation, generating vcf files, calling an average of 199.69 single-nucleotide variants (SNVs) per sample with 89.75% of these being annotated in dbSNP. Figures 5A-C shows boxplots of the numbers of SNPs per patients, see Figure 5A, numbers of hemizygous, heterozygous, and homozygous SNPs per patient, see Figure 5B, and numbers of SNPs for patients with low and high clinical score, see Figure 5C.

#### **Rare variants and structural properties**

SNPs are associated with disease severity in infection with SARS-CoV2. An average of approximately 220 SNPs per patient was collected and analyzed, see Figure 5A. The majority of variants were heterozygous or homozygous, and only a few were hemizygous, see Figure 5B. A slight enrichment of missense variants was observed in patients with high clinical score, see Figure 5C. Specifically, a mean of 81.3 missense SNPs was determined for patients with low clinical score versus 83.1 missense SNPs for patients with high clinical score, whereby the distribution in the latter group was broader.

The statistical significances of the variants with disease severity were calculated by the PLINK logistic regression, and the p-values were corrected for the covariates age and gender. Four SNPs with strongest association to clinical score are listed in Table 2. The SNPs are located in the genes *ACE*, *SIGLEC7*, *TMPRSS2*, and *SLC6A20*. Three of them (rs993496436, rs3787950, rs2276858) are previously known, but the variant of the gene ACE, which is located on chromosome 17 at position 61562445, is not present in the dbSNP database.

Table 2: List of SNPs with strongest association to clinical score. The p-values are not corrected for multiple testing. The size of our data set (159 patients) is too small to reach a false discover rate below 5%.

| Chr | Start | Variant allele | dbSNP ID | Gene | Occurrence mild/severe | p-value |
| --- | --- | --- | --- | --- | --- | --- |
| 17 | 61562445 | A>T | no | ACE | 1/9 | 0,002 |
| 19 | 51647798 | C>G | rs993496436 | SIGLEC 7 | 45/14 | 0,005 |
| 21 | 42866296 | T>C | rs3787950 | TMPRSS2 | 15/15 | 0,039 |
| 3 | 45813993 | G>A | rs2276858 | SLC6A20 | 10/2 | 0,045 |

The four SARS-CoV2-related genes may be risk variants, which individually, however, were not strongly associated with disease severity. The size of our data set (159 patients) was too small to reach a false discover rate below 5%. To increase power of the analysis and to identify the aggregated risk of variants on a gene level, when simultaneously considering the whole set of SNPs, we applied gene-based tests. The accumulative association of genes SIGLEC7, ACE, TMPRSS2, SELL, and SLC6A20 are listed in Table 3. The number of SNPs per gene varies and is given in the second column, “SNPs per gene”. False discovery rates (with correction for multiple testing), which were determined by GATES and VEGAS tests, are given. SIGLEC7 and ACE appear to have significant false discovery rates (p-value < 0.05 ) with 2 and 12 number of considered SNPs, respectively.

Table 3: Gene-based association test for the five SARS-CoV2-related genes SIGLEC7, ACE, TMPRSS2, SELL, and SLC6A20. The number of SNPs per gene considered for the test varies. False discovery rates (with correction for multiple testing) computed by two tests, GATES and VEGAS, are given.

| Gene | SNPs per gene | GATES | VEGAS |
| --- | --- | --- | --- |
| SIGLEC7 | 2 | 0,011 | 0,012 |
| ACE | 13 | 0,021 | 0,020 |
| TMPRSS2 | 8 | 0,220 | 0,254 |
| SELL | 6 | 0,483 | 0,613 |
| SLC6A20 | 16 | 0,534 | 0,398 |

Given the significant association of the *SIGLEC7* gene with clinical score, see Table 3, we explored the protein structure and function of the coding nonsynonymous SNP rs993496436 of this gene, hypothesizing that the SNP rs993496436 might be relevant for disease severity in infection with SARS-CoV2. The relevance of SNP rs993496436 has been previously suggested by Sharif-Askari et al. [6].

The SNP in the *SIGLEC7* gene exhibited a mutation from cytosine to guanine, causing a residue change from serine to cysteine at position 190 in the amino acid chain. Based on the corresponding UniProt entry, Q9Y286, we found six protein structures in the PDB database (1NKO, 107S, 1O7V, 2DF3, 2G5R, 2HRL) but none of them covered position 190. AlphaFold predicted a structure, AF-Q9Y286-F1, which covered the sequence from 1 to 467 and hence, included position 190 of the SNP, see Figures 5D and 5E.

AlphaFold computed a middle to low confidence score for the prediction of the structure in the region around position 190. The mutation is located in a loop region of a beta sheet. The flexibility of loop regions makes it difficult to estimate how the mutation effects structure and function of SIGLEC7. The software PolyPhen-2 predicted the mutation to be benign for both training sets HumDiv and HumVar with a score of 0.447 and 0.309, respectively, for the PolyPhen-2 report, see Figure S1 in the supplementary material. The mutation causes a change of residue from serine to cysteine. Serine and cysteine exhibit very different chemical features, so that – given the location of the amino acid change at the surface of the protein in a flexible loop region – one may speculate that the mutation may induce a significant change in structure and function of the protein.

### Discussion

Since the beginning of the pandemic, several manuscripts have been published on risk factors associated with a mild versus a severe course of infection with SARS-CoV2 [32-36]. Our limited sample size did not allow any definitive conclusions on genetic risk factors such as ABO or HLA type directly influencing clinical course. Regardless, though small in nature, our study has value in providing a dataset on patient demographics, blood and HLA type, as well as SNPs in genes of the NK cell receptor, renin-angiotensin-aldosterone and kallikrein-kinin systems with their respective clinical course. In addition, we provide a case study and protein structure analysis on a SNP in the *SIGLEC7* gene, which was significantly associated with clinical score.

Our finding of no significant association of HLA-A, -B, -C, and -DRB1 genotype with severity of infection with SARS-CoV2 Covid-19 was in accordance with the results of another study [23]. Also for HLA-DQB1, and -DPB1 genotypes, which were not studied by Schetelig et al. (2021), we identified no genotype as a major factor. As the study by Schetelig et al. (2021) corrected for sex, both age and age squared, both body mass index (BMI) and BMI squared, the intake of medication for diabetes mellitus or arterial hypertension and smoking status, a direct comparison of the results of our study and the study by Schetelig et al. (2021) is not possible. Both studies may suffer from insufficient prediction power to determine the role of HLA genotypes, as a much larger data set would be required to reach conclusive statements for the role of HLA genotypes. In contrast, several studies, including ours, discovered an association between male gender and age with more severe clinical course [31, 37, 38].

SIGLEC 7, which acts as an inhibitor of the cytotoxicity of NK cells and was found to be associated with clinical course in our study, may be involved in the clinical course of infection with SARS-CoV2 by its ability to mediate sialic-dependent binding to cells or to block signal transduction via dephosphorylation of signaling molecules [39]. Given the position of the SNP in the gene and its corresponding amino acid alteration, it is possible that the mutation may affect clinical course via structural and, consequently, functional changes in the protein. This will need to be validated in further studies.

In conclusion, our work provides SNP data in immunoregulatory genes in patients with SARS-CoV2 infection, stratified by clinical course. We present a structural analysis on the SIGLEC7 protein, in which a SNP we found to impact clinical course, is expected to alter protein structure and function. It is to be hoped that our findings may encourage further work on the largely still obscure functions of SIGLEC7 and to help predict clinical outcome in patients in SARS-CoV2 infection and possibly other diseases.

### Code Availability

Requests for materials and other correspondence should be addressed to the corresponding authors. The code for the analysis, summary statistics and figures generation can be found in https://github.com/nikatsaouni/Covid_SNP_Analysis

### Supporting information

*Raw sequencing data:*

Raw data of sequencing is available in the database BioProject under accession number SRP376127 [30], see <https://www.ncbi.nlm.nih.gov/bioproject/837053>.

*Table 1 (online version):*

For each individual participant, Table 1 lists metadata (gender, age, clinical score, ABO type, Rhesus (Rh) type, HLA-type) and genotypes of prothrombotic mutations Factor V Leiden (FVL) and Prothrombin G20210A (prothrombin). An Excel version of Table 1 is online available in the open data repository figshare, see <https://doi.org/10.6084/m9.figshare.21803928.v1>. The title of the file is “*Individual metadata for each participating patient”.*

Figure S1:

PolyPhen-2 report for SNP rs993496436 in the *SIGLEC7* gene. The prediction of SNP rs993496436 to be benign. The SNP causes a residue change from serine to cysteine at position 190 in the amino acid chain.

*Table S1:*

For each individual participant, Table S1 lists the SNPs and zygosity information (0: homozygous for the reference, 1: heterozygous, 2: homozygous for the alternate). Table S1 is also online available in the open data repository figshare, see <https://doi.org/10.6084/m9.figshare.20068868.v2>. The title of the file is “*SNPs per patient and zygosity information”.*

### Acknowledgements

This work was supported by the Goethe-Corona-Funds of the Goethe University Frankfurt to D.S.K. We acknowledge funding from the Alfons und Gertrud Kassel-Stiftung as part of the center for data science and AI and the DFG Cluster of Excellence Cardio Pulmonary Institute (CPI) [EXC 2026]. We also acknowledge funding from the consortia ACLF-I (Acute Liver Failure - Initiative) and ENABLE (Unraveling mechanisms driving cellular homeostasis, inflammation and infection to enable new approaches in translational medicine) (Hessian Ministry of the Arts and Sciences).

### Author contributions

P.L. and D.S.K designed the experiments. P.L. and S.K. performed the NGS. N.K., P.L.-J. and M.S. analyzed the NGS data and critically discussed data. A.-K.O. and I.K. analyzed the structural data. J. A. analyzed the clinical data. Y.K., M.V., S. C. provided human samples and critically discussed the data. C.G. and C. S. supervised the clinical testing (NGS and HLA). I.K., J. A., M.S. and D.S.K. supervised the research, analyzed data and wrote the manuscript. All authors critically read and approved the manuscript.

### Competing interests

The authors declare no competing interests.

### Figures

Figure 1: Experimental workflow.

Figure 2: Genes targeted in the next-generation sequencing panel and list of rare variants

Figure 3: Descriptive statistics. (A) Number of patients within age groups. (B) Number of patients versus clinical score. (C) Number of patients versus ABO blood type. (D) Number of patients versus HLA-A type. (E) Number of patients versus HLA-B type. (F) Number of patients versus HLA-C type. (G) Number of patients versus HLA-DRB1 type. (H) Number of patients versus HLA-DQB1 type. (I) Number of patients versus HLA-DPB1 type. (J) Mean clinical score versus ABO blood type. (K) Mean clinical score versus HLA-A type. (L) Mean clinical score versus HLA-B type. (M) Mean clinical score versus HLA-C type. (N) Mean clinical score versus HLA-DRB1 type. (O) Mean clinical score versus HLA-DQB1 type. (P) Mean clinical score versus HLA-DPB1 type. (J-O) The black line shows the overall mean of the clinical score of 2.81. The black bullets show the mean clinical score for patient groups. Violin plots superimposed by box plots indicate the densities of clinical scores. We found no statistical significance for any of the subgroups in J-P regarding the deviation of their mean clinical score from the overall mean clinical score of 2.81, i.e. Mann-Whitney U tests gave significance values larger than 5%.

Figure 4: Significance of age and gender. (A) Jitter plot of clinical score versus age. (B) Mean clinical score versus age group. (C) Mean clinical scores of female versus male patients of all ages. (B, C) The black line shows the overall mean of the clinical score of 2.81. The black bullets show the mean clinical score for patient groups. Violin plots superimposed by box plots indicate the densities of clinical scores. (D) Receiver operating characteristics (ROC) of a random forest prediction of hospitalization, i.e., clinical score 4-7, based on age and gender.

Figure 5: Single-nucleotide polymorphisms in patient cohort A) Number of SNPs per patient. An average of approximately 220 SNPs per patient was detected. B) Comparison of the number of SNPs with zygosity. C) Comparison of the occurrence of missense SNPs in the low versus high clinical score. D) The whole AlphaFold-predicted structure, AF-Q9Y286-F1, of the nonsynonymous SNP, rs993496436, encoding the protein sialic acid-binding Ig-like lectin 7 in human (Q9Y286). The mutated serine is illustrated in a stick-and-ball representation. It is located within the light blue-colored area in ribbon representation, exhibiting of a confidence score of 70 to 90, whereby 100 is the best score. The figure was generated using AlphaFold software. E) Magnification of the mutated serine illustrated in a stick-and-ball representation as in D). The mutated serine is located within a light blue-colored range, standing for a confidence score of 70 to 90.

### References

[1.	Ellinghaus D, Degenhardt F, Bujanda L, Buti M, Albillos A, Invernizzi P, et al. Genomewide Association Study of Severe Covid-19 with Respiratory Failure. N Engl J Med. 2020;383(16):1522-34. Epub 2020/06/20. doi: 10.1056/NEJMoa2020283. PubMed PMID: 32558485; PubMed Central PMCID: PMCPMC7315890.](https://doi.org/10.1101/2020.08.22.20179911)

[2.	Elliott L. Nations must unite to halt global economic slowdown, says new IMF head. The Guardian. 2019;8 October.](https://doi.org/10.1101/2020.08.22.20179911)

[3.	Alenquer M, Ferreira F, Lousa D, Valério M, Medina-Lopes M, Bergman ML, et al. Signatures in SARS-CoV-2 spike protein conferring escape to neutralizing antibodies. PLoS Pathog. 2021;17(8):e1009772. Epub 2021/08/06. doi: 10.1371/journal.ppat.1009772. PubMed PMID: 34352039; PubMed Central PMCID: PMCPMC8341613.](https://doi.org/10.1101/2020.08.22.20179911)

[4.	Guan WJ, Ni ZY, Hu Y, Liang WH, Ou CQ, He JX, et al. Clinical Characteristics of Coronavirus Disease 2019 in China. N Engl J Med. 2020;382(18):1708-20. Epub 2020/02/29. doi: 10.1056/NEJMoa2002032. PubMed PMID: 32109013; PubMed Central PMCID: PMCPMC7092819.](https://doi.org/10.1101/2020.08.22.20179911)

[5.	Jordan RE, Adab P, Cheng KK. Covid-19: risk factors for severe disease and death. BMJ. 2020;368:m1198. Epub 2020/03/29. doi: 10.1136/bmj.m1198. PubMed PMID: 32217618.](https://doi.org/10.1101/2020.08.22.20179911)

[6.	Saheb Sharif-Askari N, Saheb Sharif-Askari F, Mdkhana B, Al Heialy S, Alsafar HS, Hamoudi R, et al. Enhanced expression of immune checkpoint receptors during SARS-CoV-2 viral infection. Mol Ther Methods Clin Dev. 2021;20:109-21. Epub 2020/11/18. doi: 10.1016/j.omtm.2020.11.002. PubMed PMID: 33200082; PubMed Central PMCID: PMCPMC7658590.](https://doi.org/10.1101/2020.08.22.20179911)

[7.	Zhang C, Verma A, Feng Y, Melo MCR, McQuillan M, Hansen M, et al. Global patterns of genetic variation and association with clinical phenotypes at genes involved in SARS-CoV-2 infection. medRxiv. 2021. Epub 2021/07/08. doi: 10.1101/2021.06.28.21259529. PubMed PMID: 34230933; PubMed Central PMCID: PMCPMC8259910.](https://doi.org/10.1101/2020.08.22.20179911)

[8.	Bae M, Roh JD, Kim Y, Kim SS, Han HM, Yang E, et al. SLC6A20 transporter: a novel regulator of brain glycine homeostasis and NMDAR function. EMBO Mol Med. 2021;13(2):e12632. Epub 2021/01/12. doi: 10.15252/emmm.202012632. PubMed PMID: 33428810; PubMed Central PMCID: PMCPMC7863395.](https://doi.org/10.1101/2020.08.22.20179911)

[9.	Vuille-dit-Bille RN, Camargo SM, Emmenegger L, Sasse T, Kummer E, Jando J, et al. Human intestine luminal ACE2 and amino acid transporter expression increased by ACE-inhibitors. Amino Acids. 2015;47(4):693-705. Epub 2014/12/24. doi: 10.1007/s00726-014-1889-6. PubMed PMID: 25534429.](https://doi.org/10.1101/2020.08.22.20179911)

[10.	Gemmati D, Tisato V. Genetic Hypothesis and Pharmacogenetics Side of Renin-Angiotensin-System in COVID-19. Genes (Basel). 2020;11(9). Epub 2020/09/10. doi: 10.3390/genes11091044. PubMed PMID: 32899439; PubMed Central PMCID: PMCPMC7563402.](https://doi.org/10.1101/2020.08.22.20179911)

[11.	Paniri A, Hosseini MM, Akhavan-Niaki H. First comprehensive computational analysis of functional consequences of TMPRSS2 SNPs in susceptibility to SARS-CoV-2 among different populations. J Biomol Struct Dyn. 2021;39(10):3576-93. Epub 2020/05/16. doi: 10.1080/07391102.2020.1767690. PubMed PMID: 32410502; PubMed Central PMCID: PMCPMC7284145.](https://doi.org/10.1101/2020.08.22.20179911)

[12.	Arslan BA, Timucin AC. Immunotherapy approaches on innate immunity for SARS-Cov-2. Acta Virol. 2020;64(4):389-95. Epub 2020/09/29. doi: 10.4149/av_2020_401. PubMed PMID: 32985199.](https://doi.org/10.1101/2020.08.22.20179911)

[13.	Patel NG, Bhasin A, Feinglass JM, Belknap SM, Angarone MP, Cohen ER, et al. Clinical Outcomes in Hospitalized Patients with COVID-19 on Therapeutic Anticoagulants. medRxiv. 2020. doi: doi: https://doi.org/10.1101/2020.08.22.20179911](https://doi.org/10.1101/2020.08.22.20179911).

14.	infection WWGotCCaMoC-. A minimal common outcome measure set for COVID-19 clinical research. Lancet Infect Dis. 2020;20(8):e192-e7. Epub 2020/06/17. doi: 10.1016/s1473-3099(20)30483-7. PubMed PMID: 32539990; PubMed Central PMCID: PMCPMC7292605.

15.	Sivori S, Vacca P, Del Zotto G, Munari E, Mingari MC, Moretta L. Human NK cells: surface receptors, inhibitory checkpoints, and translational applications. Cell Mol Immunol. 2019;16(5):430-41. Epub 2019/02/20. doi: 10.1038/s41423-019-0206-4. PubMed PMID: 30778167; PubMed Central PMCID: PMCPMC6474200.

16.	Li H, Durbin R. Fast and accurate short read alignment with Burrows-Wheeler transform. Bioinformatics. 2009;25(14):1754-60. Epub 2009/05/20. doi: 10.1093/bioinformatics/btp324. PubMed PMID: 19451168; PubMed Central PMCID: PMCPMC2705234.

17.	McKenna A, Hanna M, Banks E, Sivachenko A, Cibulskis K, Kernytsky A, et al. The Genome Analysis Toolkit: a MapReduce framework for analyzing next-generation DNA sequencing data. Genome Res. 2010;20(9):1297-303. Epub 2010/07/21. doi: 10.1101/gr.107524.110. PubMed PMID: 20644199; PubMed Central PMCID: PMCPMC2928508.

18.	Sherry ST, Ward MH, Kholodov M, Baker J, Phan L, Smigielski EM, et al. dbSNP: the NCBI database of genetic variation. Nucleic acids research. 2001;29(1):308-11. Epub 2000/01/11. doi: 10.1093/nar/29.1.308. PubMed PMID: 11125122; PubMed Central PMCID: PMCPMC29783.

19.	Purcell S, Neale B, Todd-Brown K, Thomas L, Ferreira MA, Bender D, et al. PLINK: a tool set for whole-genome association and population-based linkage analyses. Am J Hum Genet. 2007;81(3):559-75. Epub 2007/08/19. doi: 10.1086/519795. PubMed PMID: 17701901; PubMed Central PMCID: PMCPMC1950838.

20.	Benjamini Y, Hochberg Y. Controlling the false discovery rate: a practical and powerful approach to multiple testing. Journal of the Royal Statistical Society, Series B. 1995;57(1):289-300.

21.	Liu JZ, McRae AF, Nyholt DR, Medland SE, Wray NR, Brown KM, et al. A versatile gene-based test for genome-wide association studies. Am J Hum Genet. 2010;87(1):139-45. Epub 2010/07/06. doi: 10.1016/j.ajhg.2010.06.009. PubMed PMID: 20598278; PubMed Central PMCID: PMCPMC2896770.

22.	Li MX, Gui HS, Kwan JS, Sham PC. GATES: a rapid and powerful gene-based association test using extended Simes procedure. Am J Hum Genet. 2011;88(3):283-93. Epub 2011/03/15. doi: 10.1016/j.ajhg.2011.01.019. PubMed PMID: 21397060; PubMed Central PMCID: PMCPMC3059433.

23.	Schetelig J, Heidenreich F, Baldauf H, Trost S, Falk B, Hoßbach C, et al. Individual HLA-A, -B, -C, and -DRB1 Genotypes Are No Major Factors Which Determine COVID-19 Severity. Front Immunol. 2021;12:698193. Epub 2021/08/13. doi: 10.3389/fimmu.2021.698193. PubMed PMID: 34381451; PubMed Central PMCID: PMCPMC8350391.

24.	Berman HM, Westbrook J, Feng Z, Gilliland G, Bhat TN, Weissig H, et al. The Protein Data Bank. Nucleic acids research. 2000;28(1):235-42. Epub 1999/12/11. doi: 10.1093/nar/28.1.235. PubMed PMID: 10592235; PubMed Central PMCID: PMCPMC102472.

25.	UniProt: the universal protein knowledgebase in 2021. Nucleic acids research. 2021;49(D1):D480-d9. Epub 2020/11/26. doi: 10.1093/nar/gkaa1100. PubMed PMID: 33237286; PubMed Central PMCID: PMCPMC7778908.

26.	Bienert S, Waterhouse A, de Beer TA, Tauriello G, Studer G, Bordoli L, et al. The SWISS-MODEL Repository-new features and functionality. Nucleic acids research. 2017;45(D1):D313-d9. Epub 2016/12/03. doi: 10.1093/nar/gkw1132. PubMed PMID: 27899672; PubMed Central PMCID: PMCPMC5210589.

27.	Jumper J, Evans R, Pritzel A, Green T, Figurnov M, Ronneberger O, et al. Highly accurate protein structure prediction with AlphaFold. Nature. 2021;596(7873):583-9. Epub 2021/07/16. doi: 10.1038/s41586-021-03819-2. PubMed PMID: 34265844; PubMed Central PMCID: PMCPMC8371605 filed non-provisional patent applications 16/701,070 and PCT/EP2020/084238, and provisional patent applications 63/107,362, 63/118,917, 63/118,918, 63/118,921 and 63/118,919, each in the name of DeepMind Technologies Limited, each pending, relating to machine learning for predicting protein structures. The other authors declare no competing interests.

28.	Varadi M, Anyango S, Deshpande M, Nair S, Natassia C, Yordanova G, et al. AlphaFold Protein Structure Database: massively expanding the structural coverage of protein-sequence space with high-accuracy models. Nucleic acids research. 2022;50(D1):D439-D44. doi: 10.1093/nar/gkab1061. PubMed PMID: 34791371.

29.	Gudmundsson S, Singer-Berk M, Watts NA, Phu W, Goodrich JK, Solomonson M, et al. Variant interpretation using population databases: Lessons from gnomAD. Hum Mutat. 2021. Epub 2021/12/04. doi: 10.1002/humu.24309. PubMed PMID: 34859531.

30.	Archive NSR. <http://www.ncbi.nlm.nih.gov/bioproject/837053> with SRA number: SRP376127. Dataset of single nucleotide polymorphisms of immune-associated genes in patients with SARS-CoV2 infection (2022). 2022.

31.	Gebhard C, Regitz-Zagrosek V, Neuhauser HK, Morgan R, Klein SL. Impact of sex and gender on COVID-19 outcomes in Europe. Biol Sex Differ. 2020;11(1):29. Epub 2020/05/27. doi: 10.1186/s13293-020-00304-9. PubMed PMID: 32450906; PubMed Central PMCID: PMCPMC7247289.

32.	Smith N, Possémé C, Bondet V, Sugrue J, Townsend L, Charbit B, et al. Defective activation and regulation of type I interferon immunity is associated with increasing COVID-19 severity. Nat Commun. 2022;13(1):7254. Epub 2022/11/27. doi: 10.1038/s41467-022-34895-1. PubMed PMID: 36434007; PubMed Central PMCID: PMCPMC9700809 2021, submitted by The Rockefeller University, which covers diagnosis of, susceptibility to, and treatment of viral disease and viral vaccines, including COVID-19 and vaccine-associated diseases. O.S. is an inventor on provisional patent no. US 63/020,063 entitled “S-Flow: a FACS-based assay for serological analysis of SARS-CoV2 infection” submitted by Institut Pasteur. The other authors declare no competing interests.

33.	Angulo-Aguado M, Corredor-Orlandelli D, Carrillo-Martínez JC, Gonzalez-Cornejo M, Pineda-Mateus E, Rojas C, et al. Association Between the LZTFL1 rs11385942 Polymorphism and COVID-19 Severity in Colombian Population. Front Med (Lausanne). 2022;9:910098. Epub 2022/07/08. doi: 10.3389/fmed.2022.910098. PubMed PMID: 35795626; PubMed Central PMCID: PMCPMC9251207.

34.	Gavriilaki E, Asteris PG, Touloumenidou T, Koravou EE, Koutra M, Papayanni PG, et al. Genetic justification of severe COVID-19 using a rigorous algorithm. Clin Immunol. 2021;226:108726. Epub 2021/04/13. doi: 10.1016/j.clim.2021.108726. PubMed PMID: 33845193; PubMed Central PMCID: PMCPMC8043057.

35.	Kaltoum ABO. Mutations and polymorphisms in genes involved in the infections by covid 19: a review. Gene Rep. 2021;23:101062. Epub 2021/03/04. doi: 10.1016/j.genrep.2021.101062. PubMed PMID: 33655087; PubMed Central PMCID: PMCPMC7906531.

36.	Hu J, Li C, Wang S, Li T, Zhang H. Genetic variants are identified to increase risk of COVID-19 related mortality from UK Biobank data. medRxiv. 2020. Epub 2020/11/18. doi: 10.1101/2020.11.05.20226761. PubMed PMID: 33200144; PubMed Central PMCID: PMCPMC7668757.

37.	Regina J, Papadimitriou-Olivgeris M, Burger R, Le Pogam MA, Niemi T, Filippidis P, et al. Epidemiology, risk factors and clinical course of SARS-CoV-2 infected patients in a Swiss university hospital: An observational retrospective study. PLoS One. 2020;15(11):e0240781. Epub 2020/11/14. doi: 10.1371/journal.pone.0240781. PubMed PMID: 33186355; PubMed Central PMCID: PMCPMC7665644.

38.	Scully EP, Haverfield J, Ursin RL, Tannenbaum C, Klein SL. Considering how biological sex impacts immune responses and COVID-19 outcomes. Nat Rev Immunol. 2020;20(7):442-7. Epub 2020/06/13. doi: 10.1038/s41577-020-0348-8. PubMed PMID: 32528136; PubMed Central PMCID: PMCPMC7288618.

39.	Yamakawa N, Yasuda Y, Yoshimura A, Goshima A, Crocker PR, Vergoten G, et al. Discovery of a new sialic acid binding region that regulates Siglec-7. Sci Rep. 2020;10(1):8647. Epub 2020/05/28. doi: 10.1038/s41598-020-64887-4. PubMed PMID: 32457377; PubMed Central PMCID: PMCPMC7250851.
