# Supplementary material for: Dataset of single nucleotide polymorphisms of immune-associated genes in patients with SARS-CoV-2 infection
Source: PLoS One. 2023 Nov 16;18(11):e0287725. doi: 10.1371/journal.pone.0287725 (PMC10653545; doi:10.1371/journal.pone.0287725)
Supplement: Attachment — Submitted filename: rebuttal_26_5_23.docx [file pone.0287725.s004.docx]

We would like to thank the reviewers for reading the manuscript, for the kind response and for their helpful suggestions. In the revised version, changes are highlighted in blue boldface letters.

**Reviewer #1:**

In this paper, the authors are proposed “Dataset of single nucleotide polymorphisms of immune-associated genes in patients with SARS-CoV2 infection”. The strengths of the paper are that it is well structured, the description of the related work is well done and that results are extensively compared to results of the similar research.

Response: We thank the reviewer for these positive comments.

Minor revisions:

- Authors should draw a graphical abstract of the proposed approach

Response: In the original manuscript we had provided a figure on the workflow of our study. However, in response to this reviewer’s suggestion we now also include an updated graphical abstract.

- Authors should justify the proposed approach.

Response: We agree with the reviewer and have now added sentences to this effect to the introduction (page 5).

- Proofread the entire manuscript.

Response: We agree with the reviewer and apologize for any previous typos. After making the requested changes, the manuscript has now been proofread again.

- Authors should submit dataset sample in supplementary files, and some supplementary files are not open.

Response: We apologize for this. We now include the entire dataset in supplementary files and have checked that they can be opened.

**Reviewer #2:**

- Dear authors, First of all, I congratulate you for doing this fascinating study. The article itself is well written, although it needs some corrections, as I mentioned in manuscript using track changes.

Response: We thank the reviewer for these encouraging words. We also thank you for explicitly pointing out errors and misleading sentences in the manuscript. Your work was a great help in the revision of the manuscript. We carefully proofread the final version. We made changes in the text according to your suggestions marked in the manuscript. Please see the list of changes below:

Page 15/16: The following sentence easily can be misunderstood:

"SIGLEC7 and ACE appear to have significant false discovery rates (p-value < 0.05 ) with 2 and 12 considered SNPs, respectively."

We changed the text to:

"Genes SIGLEC7 and ACE appear to have significant false discovery rates (p-value < 0.05). The significance for the gene SIGLEC7 is associated with a group of patients who presented with at least one of two SNPs. The significance for gene ACE is associated with a group of patients who presented with at least one of twelve SNPs."

Page 16: The following sentence easily can be misunderstood:

"The SNP in the SIGLEC7 gene exhibited a mutation from cytosine to guanine, causing a residue change from serine to cysteine at position 190 in the amino acid chain."

We changed the text to:

"The SNP in the SIGLEC7 gene represents a mutation from cytosine to guanine. During transcription to a chain of amino acids, this SNP causes a change from serine to cysteine at position 190."

- Please use the italic format for the name of genes and normal font for the protein’s names.

Response: We agree with the reviewer and apologize. All gene names have been italicized in capital letters, while protein names are written in capital letters and are not italicized.

- Success in your further research

Thank you!

**Reviewer #3:**

- It is necessary that the authors mention in the methods section, the program with which they analyzed the data of age, sex, blood group and HLA.

Response: We agree with the reviewer and thank him/her for raising this point. On page 13 we have now added the following text:

"Descriptive statistics of age, gender, blood group, and HLA

For the analysis of statistics we wrote Python scripts (Python version 3.7.6) in Jupyter Notebook. We used modules from the scipy package (version 1.4.1) for statistical calculations and applied ML algorithms from the scikit-learn library (version 0.22.1)."

- Likewise, to enrich the results, it is advisable to mention the clinical characteristics of the patients, mainly those who presented the 4 SNPs, as well as discuss the clinical data to reinforce the importance of the study.

Response: We agree with the reviewer and thank him/her for this excellent suggestion. We have added details to the results section on page 19/20 and have discussed these data on page 22. We also politely refer the reader to our table with the patients’ metadata.

- It is necessary to unify universal terminology, such as SARS-CoV-2, COVID-19, among others throughout the article.

Response: We agree with the reviewer. We have unified language with respect to SARS-CoV2 and other terms, as suggested by this reviewer.
